# Comparison of erector spinae plane block and tramadol on postoperative systemic immune-inflammatory response in breast cancer surgery: A prospective randomized controlled trial

**DOI:** 10.1097/MD.0000000000044466

**Published:** 2025-09-12

**Authors:** Ezgi Gülay, Ahmet Gültekin, İlker Yildirim, Cavidan Arar, Sibel Özkan Gürdal

**Affiliations:** a Department of Anesthesiology and Reanimation, Tekirdağ Namik Kemal University Faculty of Medicine, Tekirdağ, Türkiye; b Department of General Surgery, Tekirdağ Namik Kemal University Faculty of Medicine, Tekirdağ, Türkiye.

**Keywords:** breast cancer, erector spinae plane block (ESPB), stress response, systemic immune-inflammatory index (SII)

## Abstract

**Background:**

Breast cancer is the most commonly diagnosed malignancy and the leading cause of cancer-specific deaths among women. This study aimed to compare the effects of erector spinae plane block (ESPB) and systemic tramadol use as postoperative analgesia methods on the systemic immune-inflammatory index (SII) in patients undergoing breast cancer surgery.

**Methods:**

A total of 120 patients were included in the study and divided into 2 groups of 60 each based on the analgesia method: Group E (receiving ESPB) and Group T (receiving tramadol). SII values were measured and analyzed in both preoperative and postoperative periods.

**Results:**

In Group T, a significant increase of 1211.78 ± 1345.34 units in postoperative SII measurements compared to the preoperative period was observed (*P* < .01). In Group E, a significant decrease of 498.36 ± 786.25 units in postoperative SII measurements compared to the preoperative period was noted (*P* < .01).

**Conclusion:**

ESPB was found to provide more effective postoperative analgesia by reducing opioid consumption and demonstrated beneficial effects on inflammatory markers.

## 1. Introduction

Breast cancer ranks among the most frequently diagnosed malignancies in women and necessitates a multidisciplinary treatment strategy encompassing surgical intervention, radiotherapy, and chemotherapy.^[1]^ Surgical intervention in breast cancer aims to remove cancerous tissue. The choice of surgical approach in breast cancer is influenced by multiple factors, including the cancer’s type and stage, as well as the patient’s general health status.^[2]^ Postoperative pain following breast surgery is a frequently encountered complication and is commonly described as pain and burning sensations involving the chest, axilla, and ipsilateral upper extremity.^[3]^

The tumor microenvironment is a potent source of inflammation and may influence tumor cell proliferation, invasion, migration, and metastasis. The release of stress hormones (e.g., catecholamines, cortisol) and inflammatory mediators (e.g., interleukins, tumor necrosis factor-α) triggered by tumor surgery increases the risk of postoperative infection, delays wound healing, and may lead to multi-organ dysfunction syndrome, as well as increased morbidity and mortality.^[4]^

A recent review highlights that tramadol exhibits immunomodulatory and anti-inflammatory effects, distinguishing it from typical opioids that suppress immune function. Specifically, tramadol preserves or enhances natural killer cell activity – key for anticancer immunity – and sustains cytokine signaling pathways involved in defense.^[5]^ The analgesic effect of ultrasound-guided ESP block is significant after radical mastectomy. There are few adverse reactions and few effects on immune function, and it can promote the postoperative recovery of patients.^[6]^ Erector spinae plane block (ESPB), reduces opioid requirement and surgical stress by providing regional anesthesia. Animal models and clinical inferences support that regional blocks may attenuate surgical‑induced pro‑inflammatory mediators (e.g., IL‑6, TNF‑α, vascular endothelial growth factor [VEGF]), potentially impacting postoperative tumor spread and inflammatory index.^[7]^

In previous studies, venous blood parameters such as platelet, monocyte, lymphocyte, and neutrophil counts, as well as their derived ratios – such as platelet-to-lymphocyte ratio (PLR), neutrophil-to-lymphocyte ratio (NLR), and monocyte-to-lymphocyte ratio – have been found to be significant in the diagnosis, treatment, management, and prognostic evaluation of breast cancer patients.^[8]^ Recent studies have shown that the systemic immune-inflammation index (SII), a prognostic marker calculated using peripheral neutrophil, lymphocyte, and platelet counts (neutrophils × platelets/lymphocytes), may better reflect the body’s inflammatory status and offer improved predictive value through the integration of inflammatory cell parameters.^[9]^ Moreover, general anesthesia combined with regional anesthesia/analgesia has been shown to improve inflammatory responses and reduce the risk of metastasis, along with the expression of VEGF and transforming grow th factor-beta, in animal models of breast cancer.^[10]^

A thorough literature review confirmed that no prior clinical studies have directly compared the effects of tramadol and ESPB on the SII in breast cancer patients. While both analgesic approaches have been studied separately, their direct impact on SII remains unexplored.

Our study therefore fills a clear gap by evaluating how these 2 different analgesic strategies – pharmacologic (tramadol) versus regional (ESPB) – affect postoperative SII, a prognostic inflammatory biomarker that integrates neutrophil, lymphocyte, and platelet counts to reflect systemic inflammatory status.

## 2. Materials and methods

### 2.1. Ethics and informed consent

This study was performed in line with the principles of the Declaration of Helsinki. Approval was granted by the Ethics Committee of Tekirdağ Namik Kemal University Faculty of Medicine Clinical Research (Protocol number: 2021.84.04.02) and Clinical Trials Protocol Registration and Result System registration number (NCT06954324) were obtained. Patients were informed about the study and their written consent was obtained. The study was retrospectively registered and was conducted within 12 months.

### 2.2. Sample size and randomization

Following the acquisition of both written and verbal informed consent, this prospective, randomized, and single-blind study was carried out between May 2021, and May 2022, involving female patients, aged between 18 to 75 years, who were scheduled for elective unilateral breast surgery. An a priori power analysis conducted via G*Power revealed that a minimum of 64 participants per group would be needed to detect a moderate effect size (Cohen’s *d* = 0.5) with 80% statistical power at a .05 significance level. Although the calculated sample size to detect a moderate effect size (Cohen’s *d* = 0.5) with 80% power at a 5% significance level was 64 participants per group, only 60 participants per group were ultimately enrolled. A post hoc power analysis using G*Power (v3.1) showed that the achieved power with this sample size was approximately 77%, which is considered acceptable for exploratory clinical studies. The randomization ratio was set at 1:1 to ensure balance across both groups. Randomization was determined according to the closed envelope method and was conducted as a single-blind study. Block application was performed by an experienced anesthesiologist who was independent from the study for standardization purposes. The expert patient groups who would perform the postoperative follow-up of the patient made the evaluation without his/her knowledge.

### 2.3. Inclusion criteria

Female patients, aged between 18 and 75 years, who had American Society of Anesthesiologists (ASA) physical status I-II-III, and underwent elective, planned unilateral breast cancer surgery under general anesthesia, participated in the study voluntarily, and signed the informed consent form were included in the study.

### 2.4. Exclusion criteria

Patients were excluded from the study if they were pregnant or in the postpartum period; if bilateral mastectomy was planned; if they had a hematological malignancy, an active infection at the planned block site, a history or diagnosis of coagulopathy, multiple organ failure, or were scheduled for emergency surgery; if they had a history of chronic pain or opioid use; if they had neurological or psychiatric conditions affecting pain perception; if they refused regional anesthesia; or if they had a known allergy to local anesthetics.

### 2.5. Intervention

The patients were categorized into 2 distinct groups based on the postoperative analgesia method to be administered prior to general anesthesia: Group T (n = 60), who received intravenous tramadol, and Group E (n = 60), who received an ESPB. For both groups, demographic data (age, height, weight, BMI [Body Mass Index]), presence of neoadjuvant chemotherapy, duration of surgery, and American Society of Anesthesiologists score were recorded. Preoperative blood samples were collected for complete blood count, and the SII was calculated and documented based on these values.

Patients in Group E were taken to the regional anesthesia room and received ESPB before the operation. All patients were monitored preoperatively with electrocardiography, pulse oximetry (SpO_2_), and noninvasive blood pressure; the obtained vital signs (heart rate [HR], SpO_2_, and blood pressure) were recorded.

**Figure 1.**
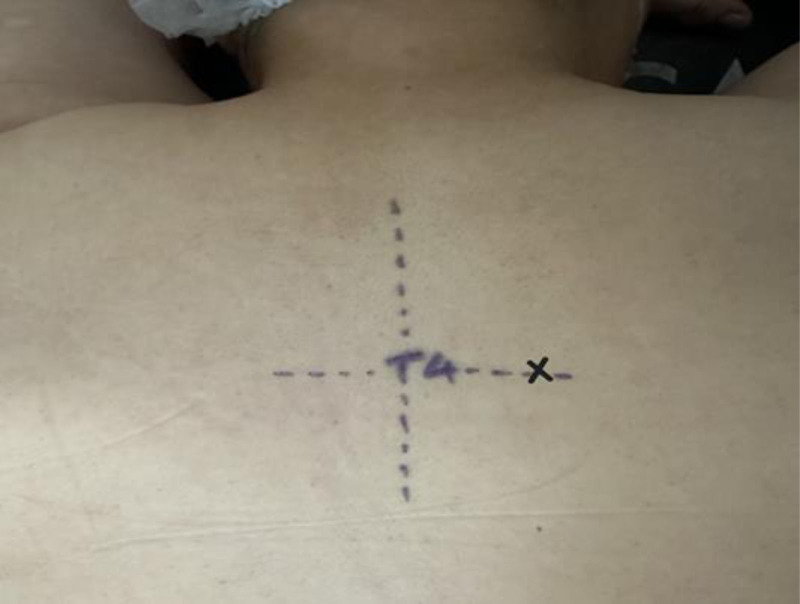
Preparation for the ESPB procedure prior to block administration, T4: Thoracic vertebra spinous process landmark, X: ESPB application point. ESPB = erector spinae plane block.

To ensure standardization, ESPB was performed by an experienced anesthesiologist, who was not involved in the study. The ultrasound device (Esaote MyLabX7, UK) with a linear probe covered in a sterile sheath and conductive gel was used. The T4 vertebral level was selected for the block. After placing the patients in the prone position and preparing the skin with 10% povidone-iodine, the spinous process of the fourth thoracic vertebra was marked with a sterile surgical skin marker (Fig. 1).

**Figure 2.**
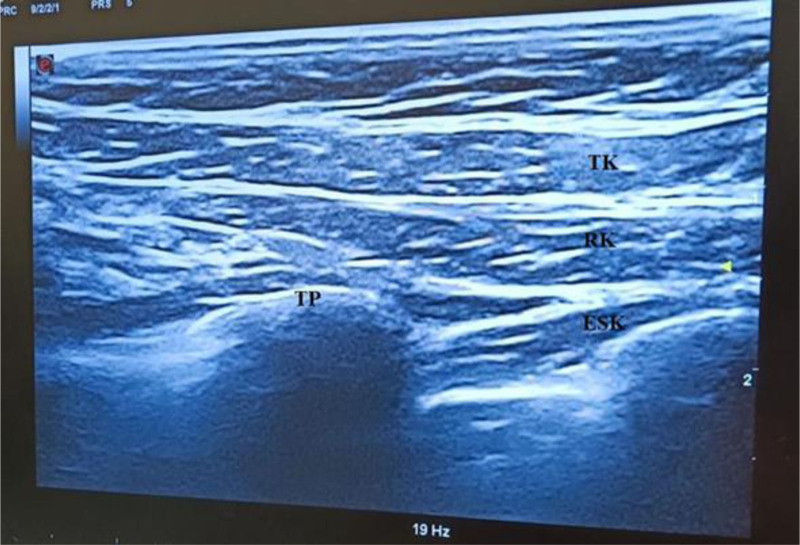
Sonoanatomy of ESPB. TK = trapezius muscle, RK = rhomboid muscle, ESK = erector spinae muscle, TP = transverse process, ESPB = erector spinae plane block.

With ultrasound guidance, after identifying the spinous process, the probe was shifted 3 cm laterally toward the side of the surgery. Sequentially, the trapezius, rhomboid major, and erector spinae muscles were visualized from superficial to deep, along with the transverse process and pleura. Following this, 2.5 mL of 2% lidocaine was administered at the site planned for peripheral nerve block needle insertion (Fig. 2).

A 22-gauge, 50 mm peripheral nerve block needle (Stimuplex Ultra 360® Braun, Germany) was advanced in a cranial-to-caudal direction using the in-plane technique. The needle was guided through the erector spinae muscle until it touched the transverse process, then was slightly retracted. Following hydrodissection to confirm the correct location, 30 mL of 0.25% bupivacaine was administered. During the injection, aspiration was performed every 5 mL to avoid inadvertent intravascular administration.

Intraoperative monitoring included routine electrocardiography, HR, noninvasive blood pressure, and SpO_2_. General anesthesia was initiated with intravenous propofol (2–3 mg/kg), fentanyl (1–2 mcg/kg), and rocuronium (0.6 mg/kg), following preoxygenation with 100% oxygen for 3 minutes. Orotracheal intubation was performed after muscle relaxation. Anesthesia was maintained using a fresh gas flow of 2 L/min with a 50% oxygen–50% air mixture and sevoflurane (MAC 1.0–1.1). In the tramadol group (Group T), 100 mg of tramadol was administered intravenously after intubation. At the end of the surgery, neuromuscular blockade was reversed using intravenous atropine (0.01 mg/kg) and neostigmine (0.03 mg/kg). Extubation was performed once the spontaneous breathing and muscle contraction had returned.

Following surgery, patients were transferred to the recovery unit, where their visual analogue scale (VAS) scores were regularly evaluated. Their HR and oxygen saturation were continuously monitored, while blood pressure was checked noninvasively at 5-minute intervals. Once patients were alert, cooperative, and hemodynamically stable with a Modified Aldrete score ≥9, they were moved to the general surgery ward. Blood samples were collected from each patient at the second postoperative hour. Using hemogram parameters, NLR, PLR, and SII were calculated and documented. VAS scores were also recorded at 0, 2, 4, and 6 hours postoperatively for both groups.

### 2.6. Statistical analysis

In the present study, statistical evaluations were performed using the SPSS 2027 software (IBM Corp., Armonk). Descriptive statistics such as mean, standard deviation, median, and the minimum and maximum values were used to summarize quantitative variables, while categorical variables were reported as frequencies and percentages. Normality of the numerical data was assessed using the Shapiro–Wilk test, supported by Box Plot graphics. For intergroup comparisons, the Student *t* test was used when data followed a normal distribution, whereas the Mann–Whitney *U* test was applied for non-normally distributed data. Repeated Measures analysis was employed to assess changes over time within groups for normally distributed data, and Bonferroni correction was used for post hoc pairwise comparisons. For 2 time-point comparisons, Paired Samples *t*-test was used if normality was met, otherwise the Wilcoxon Signed Rank test was preferred. Although repeated measures analysis is typically used for evaluating changes across 3 or more time points, it was not employed here due to the presence of only 2 time points for inflammatory markers (SII, NLR, and PLR). In this case, appropriate paired tests were applied instead. Bonferroni correction was implemented for pairwise comparisons of primary inflammatory markers (SII, NLR, and PLR) to adjust for multiple testing. Given the limited number of comparisons, this method was deemed sufficient to control the family-wise error rate. However, broader correction approaches (e.g., Holm-Bonferroni or FDR) may be considered in future studies with a larger number of outcomes. Categorical variables were compared using the chi-square test or the Fisher Freeman Halton test where applicable. Additionally, there were no missing data for key variables such as SII, NLR, PLR, or VAS pain scores. Consequently, all analyses were conducted on complete-case data, and no imputation methods were required. Statistical significance was determined at *P* < .05 with a 95% confidence interval. The study was retrospectively registered at ClinicalTrials.gov under the identifier “NCT06954324.”

## 3. Results

### 3.1. Participant characteristics

In this study conducted at Tekirdağ Namik Kemal University Hospital, a total of 120 female patients aged between 18 and 75 who underwent unilateral breast surgery after 33 patients did not meet the inclusion criteria were randomly divided into 2 groups according to the postoperative analgesia method adopted: those who received tramadol (Group T, n = 60) and those who received ESPB (Group E, n = 60). There were no statistically significant variations observed between the groups based on their demographic profiles (Table 1).

**Table 1:** Comparison of demographic data between groups.

Median (min–max)		Group T (n = 60)	Group E (n = 60)	*P*
Age (yr)		52.5 (26–75)	54.5 (31–75)	.661*
Weight (kg)		76 (47–109)	75 (48–103)	.059*
Length (cm)		160 (150–173)	160 (153–171)	.092*
BMI (kg/m^2^)		28.7 (18.4–40.2)	27.2 (20.1–39.1)	.132*
Duration of surgery (min)		97.5 (40–235)	95 (35–240)	.062*
ASA	ASA I	1 (1.7)	0 (0.0)	.763†
	ASA II	54 (90.0)	53 (88.3)	
	ASA III	5 (8.3)	7 (11.7)	
Median (min–max)		Group T (n = 60)	Group E (n = 60)	*P*
Neoadjuvant CT	Negative	39 (65.0)	32 (53.3)	.194‡
	Positive	21 (35.0)	28 (46.7)	
Sentinel lymph node	Negative	38 (63.3)	43 (71.7)	.330‡
	Positive	22 (36.7)	17 (28.3)	

ASA = American Society of Anesthesiologists, BMI = body mass index, CT = chemotherapy.

* Student *t* test.

† Fisher Freeman Halton test.

‡ Pearson chi-square.

### 3.2. Inflammatory marker outcomes

Postoperative NLR measurements in Group T were found to be significantly higher than those in Group E (*P* < .01). The increase in NLR from the preoperative to postoperative period was also statistically significant in both groups: 5.49 ± 4.78 units in Group T (*P* < .01) and 2.10 ± 4.17 units in Group E (*P* < .01).

Postoperative PLR measurements in Group T were significantly higher than those in Group E (*P* < .05). The increase in PLR from the preoperative to postoperative period was statistically significant in both groups: 76.10 ± 109.11 units in Group T (*P* < .01) and 39.77 ± 99.03 units in Group E (*P* < .01).

In Group T, the increase in SII measurements from the preoperative to postoperative period was statistically significant at 1211.78 ± 1345.34 units (*P* < .01). In contrast, Group E showed a statistically significant *decrease* in SII from the preoperative to postoperative period, with a reduction of 498.36 ± 786.25 units (*P* < .01; (Table 2).

**Table 2:** Comparison of NLR, PLR, and SII values according to postoperative analgesia method.

		Group T (n = 60)	Group E (n = 60)	*P* †
NLR				
Preoperative period	Median (min–max)	2.3 (0.6–8.8)	2.1 (0.6–18.9)	.255
Postoperative period	Median (min–max)	7.7 (1.6–22.7)	3.8 (0.7–18.8)	.001**
	*P* ‡	.001**	.001**	
∆	Mean ± SD	5.49 ± 4.78	2.10 ± 4.17	.001**
PLR				
Preoperative period	Median (min–max)	142.8 (67.8–431)	146.4 (59.8–367.9)	.879
Postoperative period	Median (min–max)	199.9 (65.2–533.9)	155.6 (78.4–547.5)	.010*
	*P* ‡	.001**	.001**	
∆	Mean ± SD	76.10 ± 109.11	39.77 ± 99.03	.025*
SII				
Preoperative period	Median (min–max)	561.2 (131–2340)	575.8 (134.5–5837)	.931
Postoperative period	Median (min–max)	1616.3 (339–7153.7)	159.8 (78.4–1489.6)	.001**
	*P* ‡	.001**	.001**	
∆	Mean ± SD	1211.78 ± 1345.34	−498.36 ± 786.25	.001**

NLR = neutrophil/lymphocyte ratio, PLR = platelet/lymphocyte ratio, SD = standard deviation, SII = systemic-immune inflammatory index.

† Mann–Whitney *U* test.

‡ Wilcoxon Signed Rank test.

** *P*<.01, statistically significant.

* *P*<.05, statistically significant.

### 3.3. Postoperative pain scores

The VAS scores at the 0th hour in Group T were found to be significantly higher than those in Group E (*P* < .01). Similarly, the VAS scores at the 2nd, 4th, and 6th hours in Group T were significantly higher than the corresponding scores in Group E (*P* < .01; Table 3).

**Table 3:** Comparison of VAS scores according to postoperative analgesia method.

Hour		Postoperative analgesia method		*P* *
		Group T (n = 60)	Group E (n = 60)	
0th	Mean ± SD	5.47 ± 1.55	4.72 ± 1.22	.004**
	Median (min–max)	5 (2–8)	5 (2–7)	
2nd	Mean ± SD	4.68 ± 1.33	3.60 ± 1.11	.001**
	Median (min–max)	5 (2–7)	3 (1–6)	
4th	Mean ± SD	4.22 ± 1.52	2.88 ± 0.90	.001**
	Median (min–max)	4 (2–8)	3 (1–5)	
6th	Mean ± SD	3.58 ± 1.27	2.52 ± 0.83	.001**
	Median (min–max)	3 (2–6)	2.5 (1–4)	
	*P* †	.001**	.001**	
∆				
0th–2nd	Mean ± SD	−0.78 ± 1.39	−1.12 ± 1.21	.164
	*P* ‡	.001**	.001**	
0th–4nd	Mean ± SD	−1.25 ± 1.13	−1.83 ± 1.01	.003**
	*P* ‡	.001**	.001**	
0th–6th	Mean ± SD	−1.88 ± 1.17	−2.20 ± 0.90	.098
	*P* ‡	.001**	.001**	

SD = standard deviation, VAS = visual analogue scale.

* Student *t* test.

† Repeated measures test.

‡ Bonferroni test.

** *P*<.01, statistically significant in footnote.

No adverse events such as postoperative nausea and vomiting, respiratory depression, pruritus, or block-related complications (e.g., hematoma, local anesthetic toxicity, or block failure) were observed in either group. Furthermore, no patients required postoperative rescue analgesia during the first 2 hours following surgery, which aligns with the timing of biochemical follow-up and early VAS evaluations.

## 4. Discussion

In breast cancer surgery, multimodal analgesia techniques administered in addition to general anesthesia have been shown not only to increase the effectiveness of analgesia but also to play an important role in the regulation of the immune response.^[11,12]^ Regional anesthesia techniques applied for postoperative pain management reduce the need for opioids, reduce the occurrence of nausea and vomiting after surgery, provide optimal pain control, enable early rehabilitation, and reduce the incidence of chronic pain.^[13]^ It is believed that the mechanisms of cancer immune regulation – elimination, equilibrium, and escape – can be triggered by inflammatory cytokines released as part of the surgical stress response, leading to tumor progression and metastasis.^[14]^

In our literature review, Buckley et al^[15]^ reported that, in women undergoing primary breast cancer surgery, natural killer cell antitumor activity was less preserved in those receiving anesthesia with a combination of sevoflurane and opioid analgesia compared to those receiving a combination of propofol and paravertebral block. In the research report published by O’Riain et al,^[16]^ 30 women undergoing mastectomy were prospectively and randomly assigned to receive either general anesthesia with postoperative opioid analgesia (a 0.1 mg/kg morphine bolus followed by patient-controlled infusion) or general anesthesia combined with paravertebral block (72-hour infusion). Venous blood samples were collected preoperatively and at the 4th and 24th postoperative hours to measure serum glucose, cortisol, C-reactive protein, VEGF, and prostaglandin E2 (PGE2) levels. While there was no significant difference between the groups in VEGF and PGE2 levels, which are markers of tumor angiogenesis, the group receiving the paravertebral block showed significantly lower levels of plasma glucose, cortisol, and C-reactive protein, indicating an inhibition of the surgical stress response.

In addition to our literature review, we compared the effects of 2 different postoperative analgesia methods on the SII, which has predictive value in immune response and tumor prognosis. Sixty patients undergoing breast cancer surgery received an ESPB (Group E) before general anesthesia as a regional anesthesia technique for postoperative analgesia, while another group of sixty patients received intraoperative tramadol after general anesthesia (Group T).

When we examined the effect of ESPB on postoperative SII values compared to tramadol, we found that the increase in SII was significantly lower in patients who received ESPB (*P* < .01; Table 2). In Group T, the postoperative increase in SII compared to preoperative values was statistically significant, with an average increase of 1211.78 ± 1345.34 units (*P* < .01). In Group E, the postoperative decrease in SII compared to preoperative values was also statistically significant, with an average decrease of 498.36 ± 786.25 units (*P* < .01). The observed reduction in postoperative SII levels in the ESPB group may be attributed to several mechanisms. Effective analgesia during the perioperative period can reduce immunosuppressive responses and preserve immune function. Peripheral nerve blocks, such as ESPB, inhibit the transmission of nociceptive signals, thus decreasing postoperative pain and associated stress responses. ESPB has been shown to block the thoracic nerve, long thoracic nerve, and dorsal thoracic rami, which may contribute to attenuated release of pain-related mediators and systemic inflammatory responses in patients undergoing breast cancer surgery. These effects collectively support the potential of ESPB to modulate the postoperative inflammatory environment.^[6]^ Since our evaluation was based on the rate of change in preoperative and postoperative values according to the type of postoperative analgesia administered, no cutoff value was determined for SII in this study.

Studies have shown that NLR, PLR, and lymphocyte-to-monocyte ratio, which are calculated based on complete blood count results, can serve as clinical prognostic indicators for various cancer types by reflecting systemic inflammatory responses.^[17,18]^ Recent studies suggest that the choice of postoperative analgesia method used during the perioperative period may influence the prognosis of cancer patients, and the SII has been used as a prognostic marker in many of these studies.^[19,20]^

Previous research has demonstrated that the immune response is associated with tumor metastasis as well as clinical and pathological progression of cancer. In our study, we also observed favorable results consistent with previous research by comparing the effects of analgesic techniques on SII, an immune parameter that is both inexpensive and easy to calculate.

It is worth noting that there were certain limitations in our study. Firstly, it was a single-center design and homogeneous patient groups could not be fully achieved due to variations in the surgical techniques applied. Additionally, the study was retrospectively registered, which may raise concerns regarding protocol transparency and the pre-specification of outcomes. Another important limitation is the short follow-up duration; SII, NLR, and PLR values were measured only at 2 hours postoperatively to assess the early inflammatory response. Although this time point was chosen based on prior evidence suggesting early immune activation following surgical injury, systemic inflammation may peak at later time points (e.g., 24–48 hours), and the lack of serial measurements limits our understanding of the full temporal dynamics. Similarly, pain scores were assessed only up to 6 hours postoperatively, potentially overlooking delayed analgesic effects or rebound pain phenomena. Moreover, while the sample size was calculated to detect a moderate effect size with adequate power, it may be insufficient to identify rare adverse events or subgroup-specific differences. Finally, long-term effects such as chronic pain, cancer recurrence, or survival were not evaluated. Future multicenter studies with prospective registration, standardized surgical protocols, extended follow-up periods, and serial biomarker assessments are likely to provide a more comprehensive and generalizable evaluation of the clinical and immunologic effects of ESPB in breast cancer surgery.

We believe that the preferred method of analgesia may influence postoperative SII, which in turn may affect the postoperative immune response and ultimately play a role in cancer prognosis.

## Acknowledgments

We want to acknowledge the support of patients and healthcare professionals who made this study possible. During the preparation of this manuscript, a language editing assistance was provided using ChatGPT (OpenAI), an AI language model, and Berfu Gülay. The final content was reviewed and approved by the authors. We would like to thank Emire Bor for her valuable support in statistical consultancy throughout the study.

## Author contributions

**Conceptualization:** Ezgi Gülay, Ahmet Gültekin.

**Data curation:** Ezgi Gülay.

**Formal analysis:** Ezgi Gülay, Ahmet Gültekin, Cavidan Arar.

**Investigation:** Ezgi Gülay.

**Methodology:** Ahmet Gültekin, Sibel Özkan Gürdal.

**Supervision:** İlker Yildirim, Cavidan Arar.

**Validation:** İlker Yildirim.

**Writing – original draft:** Ezgi Gülay, Ahmet Gültekin.

**Writing – review & editing:** Ezgi Gülay, Ahmet Gültekin.
